# Tumor Targeted Multifunctional Magnetic Nanobubbles for MR/US Dual Imaging and Focused Ultrasound Triggered Drug Delivery

**DOI:** 10.3389/fbioe.2020.586874

**Published:** 2020-12-07

**Authors:** Zhen Jin, Jinlong Chang, Peipei Dou, Shang Jin, Min Jiao, Heyun Tang, Wenshuai Jiang, Wu Ren, Shaohui Zheng

**Affiliations:** ^1^College of Biomedical Engineering, Xinxiang Medical University, Xinxiang, China; ^2^School of Medical Imaging, Xuzhou Medical University, Xuzhou, China

**Keywords:** multifunctional magnetic nanobubbles, US/MR imaging, tumor targeting, focused ultrasound controlled release, enhanced cellular uptake

## Abstract

The development of multifunctional nanoplatforms that are safe and have multiple therapeutic functions integrated with dual- or multi-imaging modality is one of the most urgent medical requirements for active cancer therapy. In our study, we prepared multifunctional magnetic nanobubbles (MF-MNBs) by co-encapsulating superparamagnetic iron oxide nanoparticles (SPIONs) and doxorubicin into polylactideco–glycolide–polyethylene glycol–folate (PLGA-PEG-FA) polymer-based nanobubbles for tumor-targeted ultrasound (US)/magnetic resonance (MR) imaging and focused ultrasound (FUS)-triggered drug delivery. Hydrophobic SPIONs were successfully embedded into MF-MNBs by a typical double emulsion process. The MF-MNBs were highly dispersed with well-defined spherical morphology and an average diameter of 208.4 ± 12.58 nm. The potential of MF-MNB as a dual-modal contrast agent for US and MR imaging was investigated via *in vitro* study, and the MF-MNB exhibits promising US/MR contrast ability. Moreover, tumor targeting ability was further enhanced by folate conjugation and assessed through *in vitro* cell test. Furthermore, FUS, as a non-invasive and remote-control technique, was adopted to trigger the release of doxorubicin from MF-MNB and generate the sonoporation effect to enhance drug release and cellular uptake of MF-MNBs. The 4T1 cell viability was significantly decreased by FA ligand-receptor-mediated targeting and FUS sonication. In addition, the developed MF-MNB also exhibits enhanced accumulation in tumor site by FA ligand-receptor-mediated tumor targeting, in which the accumulation of MF-MNB was further enhanced by FUS sonication. Hence, we believe that the MF-MNB could be a promising drug nanocarrier for US/MR-guided anticancer drug delivery to improve cancer treatment efficacy.

## Introduction

Cancer has been one of the leading causes of death in the world according to world cancer statistics (Zeng et al., 2019). Conventionally, cancer treatment mainly includes chemotherapy, radiotherapy, and clinical surgery. Although these therapeutic approaches can significantly inhibit cancer growth, they could be accompanied by severe side effects, such as systemic toxicity, recurrence, or physiologic dysfunction (Kievit and Zhang, 2011). Recently, the rapid development of nanotechnology has significantly influenced the strategy of cancer diagnosis and treatment. Various nanomaterials have been proposed as therapeutic drug carriers to achieve efficient therapy by enhancing the availability of therapeutic drugs and reducing side effects (Shi et al., 2017). Nanocarrier accumulation in solid tumor is generally based on the enhanced permeability and retention (EPR) effect, also called passive targeting (Maeda, 2010). However, the intravenously administered nanocarriers faced with physical and biological barriers [for example, intratumoral pressure, shear forces and binding-site effect, mononuclear phagocytic system (MPS) sequestration, and renal clearance] expected therapeutic outcomes that occur only if the nanocarriers could overcome the barriers to accumulate in tumor at a sufficiently high dosage (Rosenblum et al., 2018). Generally, the passive accumulation efficiency is highly heterogeneous and depends on the nanocarrier’s property, tumor size, location, and progress state (Blanco et al., 2015). Therefore, extensive efforts have been devoted to develop multifunctional nanocarriers that combine therapeutic and diagnostic capabilities in a single probe to achieve imaging-guided cancer therapy (Kalidoss et al., 2019; Wang et al., 2020), which enables one to monitor the distribution and accumulation of nanocarriers and evaluate the treatment efficiency during and after therapy.

Furthermore, for accurate cancer imaging, it is meaningful to design multifunctional nanocarriers that afford dual- or multi-modal imaging as each imaging modality has its own limitations and advantages (Ju et al., 2019). Ultrasound (US) has been extensively used as a diagnostic tool in clinical application because of its non-invasiveness, high biosafety, and low cost. Moreover, US is also a promising external stimulus to trigger the drug release and enhance cellular uptake compared with other external stimuli, such as near-infrared (NIR) light, magnetic field, and electric field (Mura et al., 2013), because US is easy to focus and penetrates deeply into soft tissue in a non-invasive manner. However, US has also exhibited poor deep tissue discrimination ability ascribed to its low image resolution (Jain et al., 2018). In contrast, magnetic resonance (MR) imaging is a powerful, non-invasive technique to obtain real-time images containing morphological and functional information with high resolution (Li et al., 2015). Gas-filled microbubbles (MBs) and superparamagnetic iron oxide nanoparticles (SPIONs) are clinically used contrast agents for US and MR imaging, respectively (Geers et al., 2012; Li et al., 2015). For MR imaging, there are numerous promising design strategies for drug nanocarriers that can also act as MRI contrast agent (Zhu et al., 2017). However, effective ultrasonography generally requires enough large-sized MBs to obtain sufficient resonance reflection at the diagnostic US frequency. On the other hand, for effective anticancer drug delivery, it is essential to develop nanosized contrast agents that can efficiently penetrate through the leaky vasculature and accumulate inside tumors (the gaps range in tumor vasculature is 600 to 800 nm) (Blanco et al., 2015). Therefore, it remains a critical challenge to integrate the advantage of nanosized contrast agents with long circulation and EPR effect-induced accumulation in tumor sites and micrometer-sized MBs with effective US responsibility (Cui et al., 2019).

In the past decade, extensive efforts have been devoted to design nanocarriers to handle the above critical problem. For example, M.A. Malvindi et al. (2011) presented a magnetic iron oxide nanoparticle decorated silica nanocomposites that can enhance US imaging and T_2_-weighted MR imaging signals. The authors found that the nanocomposites exhibited significant US backscatter enhancement and heightened T_2_-weighted MR signal intensity. However, the low biodegrability, unclear biological effect, and long-term toxicity should be further addressed for clinical application. H. Tang et al. fabricated a liquid-formed PFH and SPIO-loaded PLGA nanoparticles for US and MR imaging. The phase of PFH could be changed from liquid to gas when the environment temperature elevated to 56°C by HIFU sonication (Tang et al., 2018). Therefore, the enhanced US imaging signal is only effective in the area upon HIFU sonication. Moreover, Prabhakar and Banerjee (2019) also illustrated submicron-sized nanobubble-paclitaxel liposome for US imaging and anticancer drug delivery. However, the soft and unstable structure of the liposome will result in easy dissolution of gas. Thus, the further modification of nanomaterials for MR imaging will be limited due to the complex structure. Recent studies have reported that the presence of SPIO nanoparticles in the bubble shell can alter the surface tension of bubbles, boost the acoustic impedance, and enhance the detectable backscatter, leading to enhanced US imaging signal (Huang et al., 2013). Sun et al. reported magnetite-loaded polypeptide-PLGA-iron MBs for dual-modal US/MR imaging for prostatic cancer (Sun et al., 2016). However, when used in diagnostic US, the MBs easily become trapped in the blood pool and are limited to the vascular system after intravenous injection attributed to their micro-scaled size, hindering their application in tumor-targeted therapy and imaging (Zhang et al., 2014). The ideal nanocarrier for US/MR imaging should be biocompatible and biodegradable, have a long circulation lifetime, and be smaller than a certain size to retain EPR effect and active tumor targeting.

Here, in this study, we presented novel multifunctional magnetic nanobubbles (MF-MNBs) that are biocompatible and biodegradable, and combined them with the functions of a MR/US dual image contrast agent, focused ultrasound (FUS)-triggered drug release, and FA ligand-receptor-mediated active tumor targeting, which was prepared by a one-pot double emulsion process. First, we synthesized the PLGA-PEG-FA copolymer using reported carbodimide reaction, in which the PEG chain could prolong the circulation half-life in the bloodstream and the folate ligand could enable active tumor targeting. Then, SPIO nanoparticles were fabricated with a conventional chemical co-precipitated method with slight modification. Subsequently, MF-MNBs were prepared by co-encapsulating anticancer drugs DOX and SPIO in PLGA-PEG-FA nanobubbles with a one-pot double emulsion method and later filling them with perfluorocarbon gas. The MF-MNB exhibits promising US and MR image contrast capability due to the densely aggregated SPIO nanoparticles in the bubble shell to provide excellent magnetic property and enhance US signal. Moreover, significantly faster and higher DOX release from MF-MNBs was achieved by FUS triggering. In addition, the cellular uptake and cytotoxicity were dramatically enhanced by FA receptor-mediated targeting and FUS sonication. Moreover, the MF-MNB exhibits enhanced accumulation in tumor site by FA receptor-mediated tumor targeting, and it was then further enhanced by FUS sonication. Therefore, we believe that the developed MF-MNB could be a promising drug nanocarrier for US/MR dual-image guided drug delivery for efficiently treat cancer.

## Materials and Methods

### Materials

Iron(II) chloride, iron(III) chloride, oleic acid, sodium hydroxide, chlorhydric acid, 4,6-diamidino-2-phenylindole (DAPI), 3-(4,5-dimethylthiazol-2-yl)-2,5-diphenyltetrazolium bromide) (MTT), polyvinylalcohol (PVA, MW = 30–70 kDa), poly(lacticco-glycolic acid) (PLGA, molar ratio of 50:50, MW = 7000–17,000), N-hydroxysuccinimide (NHS), dicyclohexyl carbodiimide (DCC), dichloromethane (DCM), dimethyl sulfoxide (DMSO), and folic acid were purchased from Sigma-Aldrich. Poly(ethylene glycol amine) (PEG-diamine, MW 3400) was received from Laysan Bio, Inc., Doxorubicin (DOX) was bought from Beijing HuaFeng United Technology Co., Ltd. Cell culture medium [Dulbecco’s modified Eagle’s medium (DMEM)], penicillin, streptomycin, and fetal bovine serum (FBS) were obtained from Gibco BRL (Annapolis, MD, United States). All chemicals were of analytical grade and used without further purification, and all aqueous solutions were prepared using ultrapure water from a Milli-Q system (Millipore, Billerica, MA, United States).

### Synthesis of PLGA-PEG-FA Polymers

The fabrication of PLGA-PEG-FA was prepared with carbodimide reaction with the following two steps: coupling of PLGA and PEG, followed by FA conjugation (Jain et al., 2012). Briefly, PLGA was activated by DCC and NHS in DCM solvent for 24 h. Then, PEG-diamine was added in the resultant solution for 12 h. The product from the abovementioned reaction step was washed and purified with ice-cold diethyl ether, followed by completely drying under vacuum. Thereafter, the final PLGA-PEG product was dissolved in DMSO and dialyzed (MWCO: 3.5 kDa) against DI water for 2 days, and the product was freeze-dried for 2 days. For FA conjugation, FA was firstly activated with DCC for 2 h. Then, the PLGA-PEG-NH_2_ dissolved in DMSO was added to activated FA solution in a dropwise manner (PLGA-PEG-NH_2_:FA:DCC = 1:2:2.5). The reaction was performed under nitrogen gas protection at room temperature for 12 h. Then, the solution was dialyzed (MWCO: 3.5 kDa) against distilled water for 48 h to remove the unreacted FA and freeze-dried to obtain the PLGA-PEG-FA product.

### Synthesis of Superparamagnetic Iron Oxide (SPIO) Nanoparticles

Hydrophobic SPIO was synthesized using a chemical co-precipitation method with slight modification (Schleich et al., 2013). Briefly, 10 mmol iron(III) chloride and 5 mmol iron(II) chloride were dissolved in 24 ml of HCl aqueous solution (1M). Then, the solution was added dropwise to an aqueous solution of NaOH (1M) under vigorous mechanical stirring for 60 min with the protection of dry nitrogen at room temperature. Then, 3 g of oleic acid was added and stirred for another 30 min at 80°C. Thereafter, the black precipitate was magnetically separated, washed thrice using anhydrous ethanol, and then dispersed in 20 ml of methylene chloride. The solution was then placed in an ultrasonic bath for 10 min and centrifuged (2000 rpm, 10 min) to remove the undispersed residue.

### Synthesis of Multifunctional Magnetic Nanobubble (MF-MNB)

MF-MNB was fabricated by a double water/oil/water (W/O/W) emulsion solvent evaporation method (Sun et al., 2012). Briefly, 1 ml (0, 10, 20, 30, 40, and 50 mg/ml) of SPIO was mixed with the organic solution of the PLGA-PEG-FA solution (100 mg of polymer in 2 ml of methylene chloride). Then, 0.3 ml of DOX solution (10 mg of DOX dissolved in 0.3 ml of deionized water) was added to the previous organic phase. Thereafter, the mixture was emulsified by sonication for 1 min at 300 W (JY92-II, Ningbo Scientz Biotechnology Co., Ltd., China). Subsequently, 10 ml of poly(vinyl alcohol) (PVA) solution (5 wt%) was added to this resultant emulsion, and the mixture was then further sonicated in an ice bath for 5 min. The resultant double emulsion was diluted in 50 ml of PVA solution (0.5 wt%) under mechanical stirring at room temperature overnight to evaporate the residual methylene chloride from the prepared MF-MNBs. Then, the MF-MNB solution was centrifuged and washed thrice with DI water to obtain the pure MF-MNBs. Finally, the MF-MNBs were freeze-dried, filled with perfluorocarbon (C_3_F_8_) gas, and stored in a freezer at 4°C for further use. Moreover, the PLGA MNB and PLGA-PEG MNB were prepared with the same process for the negative control group test. The amount of SPIO encapsulated in the MF-MNB was measured by inductively coupled plasma optical emission spectroscopy (ICP-OES), and the amount of DOX was measured using a calibration curve of DOX at a wavelength of 480 nm. The DOX loading capacity was calculated as W = W_totoal DOX_ -W_DOX_ in supernatant.

### Characterizations of MF-MNBs

The structure and the percentage of the actual molar compositions of PLGA-PEG-FA were determined by ^1^H NMR analysis and the copolymer structures were confirmed by FT-IR using KBr disk (Thermo Nicolet Nexus 670, Ramsey, MN, United States). The morphology and structure of SPIO and MF-MNB were observed via a scanning electron microscope (SEM, SS-550; Shimadzu, Kyoto, Japan) and a transmission electron microscope (TEM, JEM-2100F, JEOL Ltd, Tokyo, Japan). The mean diameter, size distribution, and zeta potential of MF-MNB were measured by a DLS method in a Zetasizer nano ZS90 analyzer (Malvern Instruments Ltd., United Kingdom). The magnetization property was further evaluated using a vibrating sample magnetometer (VSM, Lake Shore Cryotronics 7404, Westerville, OH) at room temperature.

### *In vitro* US and MR Imaging

*In vitro* US imaging was performed to evaluate the US contrast behavior of magnetic nanobubbles in 1% agarose gel mold, which was prepared with a casting method (Prabhakar and Banerjee, 2019). US images of air, degassed water, MF-MNB with different concentration of nanobubbles without SPIO loading, and MF-MNB with the same nanobubbles but different weight percent of SPIO were acquired by placing the samples in the holes of agarose phantom. An ultrasonic imaging system (alpinion medical system; E-CUBE-12R) with a 7.5-MHz US transducer was used as a transmitter and a receiver. All US images were acquired with the same instrument parameters (Mechanical Index, MI = 0.1; gain = 10 dB).

All MR imaging studies were performed on a Philips Achieva 3.0-T MRI scanner (Philips Achieva 3.0T TX, Philips Medical Systems, Netherlands). Magnetic nanobubbles were diluted to final iron concentrations of 0, 0.025, 0.05, 0.1, 0.2, 0.4, and 1.2 mM in PBS (pH 7.4) and placed in Eppendorf tubes of 1 cm in diameter. T_2_-weighted (T_2_ WI) images were obtained using the following parameters: repetition time (TR) = 72 ms, echo time (TE) = 9 ms, and slice thickness of 3.0 mm. The MRI signal intensity within the region of interest (ROI) was also measured.

### FUS System Setup for Triggering Drug Release and Cell Test

The FUS system setup for triggering drug release and cell test was similar to our previous study (Zheng et al., 2016; Jin et al., 2017). Briefly, a single element US transducer (aperture diameter: 30 mm, focal length: 29.7 mm, frequency: 925 kHz) was employed to generate US, and a function generator (33210A, Agilent, Palo Alto Ca, United States) was applied to create burst signal. A power amplifier (HAS-4014, NF Corporation, Yokohama, Japan) was used to amplify the signal from the function generator, and a self-assembled external impedance matching circuit was used to match the electric impedance of the acoustic transducer with the output impedance of the amplifier. A needle-type polyvinylidene fluoride (PVDF) hydrophone with a sensing element of 0.2 mm (Precision Acoustics; Dorchester, United Kingdom) was adopted to measure the acoustic pressure in a tank filled with degassed and distilled water. The half-maximum pressure amplitude at the focal point of FUS has a diameter of 5.6 mm. All experiments were performed in a tank containing degassed water at 37°C. The FUS irradiation was applied at a frequency of 925 kHz, a pulse length of 1000 cycles, and a pulse repetition frequency (PRF) of 20 Hz.

### FUS-triggered Drug Release

The *in vitro* DOX release from MF-MNB was investigated in the presence and absence of the FUS triggering. Briefly, 10 mg of MF-MNB with an SPIO weight percent of 17.6 wt% was dispersed in 5 ml of PBS solution (pH 7.4) and transferred to a dialysis bag (MWCO: 3500 Da). Then, the dialysis bag was placed in a sample holder in a test tube containing 50 ml of PBS in which the bottom wall is silicon membrane. After 3 min of FUS triggering, the sample was transferred to a shaking reservoir at 150 rpm at 37°C (Zhang et al., 2014). Thereafter, 1 ml of medium was removed from the samples and stored at −20°C for further analysis, and 1 ml of fresh PBS was added back to the tube to maintain a constant volume. The concentration of DOX in each sample was analyzed using a plate reader system. Then, the accumulative rates of the release DOX were calculated as a function of time. Controls were performed using the same setup and time scale, but without FUS triggering.

### *In vitro* Cellular Uptake and Cellular Imaging

Enhanced intracellular uptake of MF-MNB mediated by folic acid and FUS irradiation was quantitatively analyzed by ICP-OES according to the analytical method. A total of 2 **×** 10^4^ of 4T1 cells were seeded in 48-well plates and incubated overnight for cell attachment: a part of 4T1 cells were pretreated with a high dose of free FA for 4 h to block the FA receptors. Then, cells were incubated with MF-MNB for 4 h to allow cellular uptake, and the FUS was applied to the corresponding samples for 3 min to further enhance cellular uptake. Subsequently, cells were washed with ice-cold PBS (pH 7.4) thrice; 500 μl of radio-immunoprecipitation assay (RIPA) lysis and extraction buffer was added to each well and incubated for 30 min to allow lysate extraction from cells. The iron content in the cell lysates was analyzed by ICP-OES, and the protein content of cell lysates was measured according to the standard bovine serum albumin (BSA) assay performed.

The cellular uptake efficiency of the developed MF-MNB with FUS triggering was also evaluated. 4T1 cells were seeded on culture slides at a density of 10^5^ cells per well (1.7 cm^2^ of surface area per well) and incubated for 24 h at 37°C. Then, the medium was replaced with free DOX, blank MF-MNB, and DOX-loaded MF-MNB solutions. Then, certain groups were treated with FUS for 5 min and incubated for another 2 h at 37°C. The cells were washed with PBS (pH 7.4) and fixed with 4% formaldehyde solution for 10 min. Then, the samples were mounted after complete drying of the liquid with mounting medium with 4,6-diamidino-2-phenylindole (DAPI; H-1200, Vector Laboratories, Inc., CA, United States) to prevent fading. The slides were observed using confocal laser scanning microscopy (LSM510, Zeiss, Germany).

### Cell Cytotoxicity Test

The cytotoxicity was determined by live and dead cell detection and a standard MTT cell proliferation assay. Briefly, 4T1 breast cancer cells were seeded at a density of 1 **×** 10^4^ cell per well in 96-well plates and incubated for 24 h for cell attachment. Then, the used medium was replaced with free DOX, DOX-unloaded MN-MNB, and DOX loaded MF-MNB solutions (DOX concentration of 10 μg/ml). After 12 h, the cell wells were placed on a sample holder at 37°C and a certain group was triggered with FUS for 3 min. After further incubation for 6 h, supernatants were removed. Then, the cells were stained with calcein AM and PI, and the live and dead cells were observed with fluorescence microscope images (Nikon, United States). For the cell viability test, the cells were treated with the same drug formula mentioned above and a certain group was treated with FUS for 3 min. After further 12-h incubation, the wells were washed twice with PBS and incubated with DMEM containing MTT (5 mg/ml) for another 4 h. The MTT solution was removed and dimethyl sulfoxide (DMSO) was added to dissolve the cells for spectrum measurement. The absorbance at 570 nm was measured by a microplate reader (Thermo Scientific, Waltham, CA, United States).

### *In vivo* Biodistribution and Pathological Analysis

For *in vivo* biodistribution test, 8-week old BALB/c mice bearing 4T1 breast cancer tumors were prepared. All animal care and procedures were conducted according to the protocols approved by the Xinxiang Medical University Animal Research Committee (Xinxiang, Henan, China). For the 4T1 tumor-bearing mouse model, 1 × 10^7^ cells in 100 μl of serum free RMPI-1640 medium were subcutaneously injected onto the back side of each mouse. The mouse models were used when their tumor volumes approached 60–70 mm^3^. To determine the *in vivo* biodistribution of MF-MNB, 4T1 tumor-bearing mice were i.v. injected with MF-MNB, and a certain group was sonicated with FUS for 3 min. Then, the mice were sacrificed at 4 and 24 h. Major tissues (heart, lung, liver, spleen, kidney, and tumor) from these mice were collected and weighted. Subsequently, the tissues were disrupted by TissueRuptor (QIAGEN) and digested using 6 ml of 65% HNO_3_ and 4 ml of 30% H_2_O_2_ solution at 80°C. The digested samples were diluted with deionized water to a total volume of 25 ml. Thereafter, the iron concentration in each sample was evaluated by inductively coupled plasma optical spectrometry (ICP-OES). For the pathological analysis, the 4T1 tumor-bearing mice were sacrificed at 7 days and 21 days post injection of MF-MNB. Major organs were collected, fixed in 10% formalin solution, processed routinely into paraffin, sectioned at 8 μm, stained with hematoxylin–eosin, and examined with a digital microscope (Nikon United States).

### Statistical Analysis

One-way ANOVA was performed to evaluate the significance of multiple groups. *p* < 0.05 was selected as the significance level, and the data were indicated (^∗^) for *p* < 0.05, (^∗∗^) for *p* < 0.01, and (^∗∗∗^) for *p* < 0.001.

## Results

### Synthesis and Characterization of MF-MNB

**FIGURE 1:**
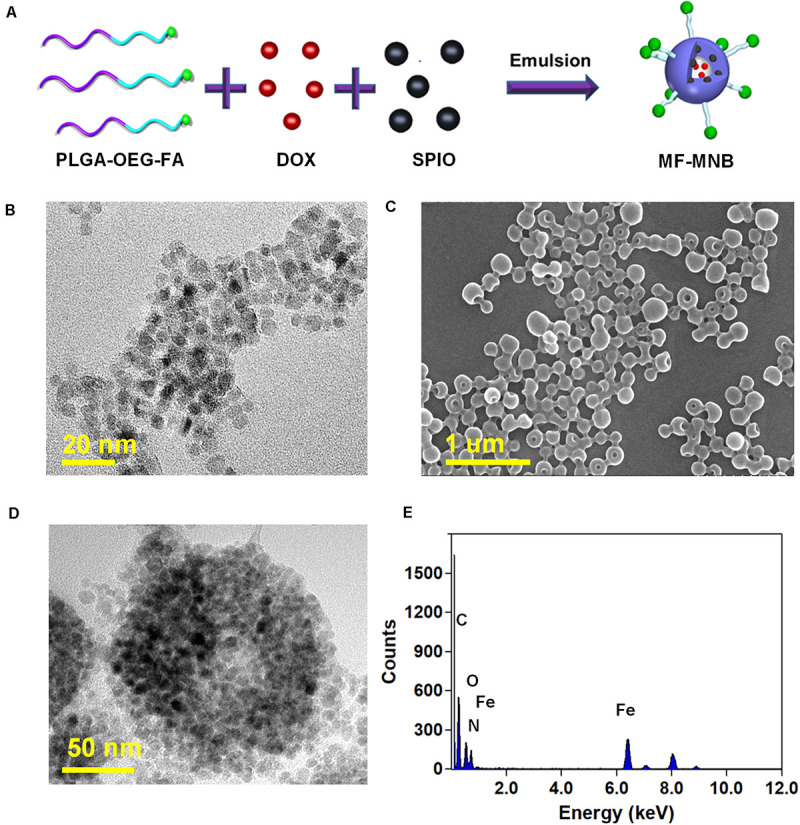
Preparation of multifunctional magnetic nanobubbles (MF-MNBs). **(A)** Schematic illustration of the fabrication process of MF-MNB. **(B)** TEM image of SPIO nanoparticles. **(C,D)** SEM and TEM image of MF-MNB. **(E)** EDS spectrum curve of Fe, O, and N elements in MF-MNB.

**FIGURE 2:**
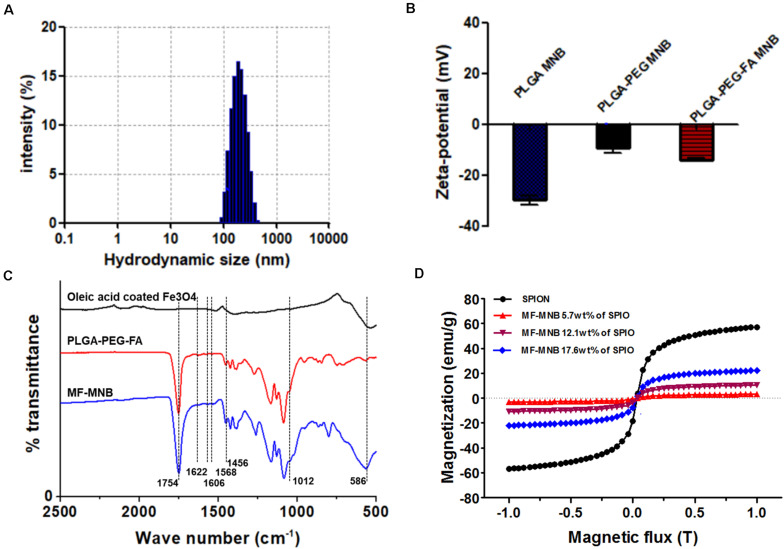
Characterization of MF-MNB. **(A)** Hydrodynamic size of MF-MNB in PBS solution (pH 7.4). **(B)** Zeta potential of PLGA MNB, PLGA-PEG MNB, and PLGA-PEG-FA MNB (MF-MNB), respectively. **(C)** Fourier transform infrared (FTIR) spectrum curve of SPIO, PLGA-PEG-FA polymer and MF-MNB, respectively. **(D)** Magnetization curve of SPIO and MF-MNB with different encapsulation concentration of SPIO.

The fabrication process of MF-MNB is illustrated in Figure 1A for the synthesis of MF-MNB. First of all, the PLGA-PEG-FA copolymer was prepared by carbodiimide reaction. The conjugate structure of PLGA-PEG-FA was confirmed by ^1^HNMR spectroscopy (Supplementary Figure 1). The principal peaks related to benzene of the folate moiety (δ = 6.7, 6.9, 7.7, and 7.9 ppm), the PEG (δ = 3.4 and 3.7 ppm), and the PLGA moiety (δ = 5.1 and 4.7 ppm) reveal the successful fabrication of the PLGA-PEG-FA copolymer. Then, SPIO nanoparticles were prepared with the traditional chemical co-precipitation method with slight modification. The size and morphology of SPIO were characterized using transmission election microscopy (TEM) (Figure 1B), and the TEM image of SPIO exhibits a spherical morphology with relatively uniform size ranging from 4 to 7 nm. Subsequently, MF-MNBs were prepared with the double emulsion method. The SEM image of MF-MNBs indicated that the MF-MNB revealed a uniform and spherical morphology without aggregation (Figure 1C). The presence of SPIO nanoparticles in the polymer shell of MF-MNB was determined by the enhanced contrast manifested as dark domains in the TEM image (Figure 1D). The polymer shell was also visible as gray areas surrounding black spots (SPIO). Moreover, the existence of Fe, O, and N elements in MF-MNB was further validated with EDS spectrum (Figure 1E). The mean hydrodynamic size of MF-MNB was 208.4 ± 12.58 nm, and the size ranged from 91.7 nm to 459 nm in PBS solution (Figure 2A). The zeta potential of PLGA-PEG MNB exhibits a higher negative value of −9.17 ± 1.95 mV than the value of PLGA MNB (−28.84 ± 1.82 mV), which may be attributed to the presence of an amine group in the PEG chains. In comparison, after FA conjugation, the value of MF-MNB was slightly decreased to −13.5 ± 0.762 mV, ascribed to the binding of FA on the amine group in the PEG chains (Figure 2B). Moreover, the synthesized MF-MNB exhibits excellent colloidal stability in PBS buffer (pH 7.4) and DMEM cell culture medium with a narrow size distribution for 24 h (Supplementary Figure 8). Then, the conjugate structure of MF-MNB was further confirmed using FTIR spectrum (Figure 2C). The strong absorption with vibration bands at 586 cm^–1^ and 1012 cm^–1^ could be attributed to Fe–O vibration mode. The peak at 1453 cm^–1^ and 1606 cm^–1^ is due to the stretching vibrations of C = C in the backbone of the aromatic ring of folic acid. The peak at 1622 cm^–1^ and 1568 cm^–1^ may be due to the presence of carbonyl (C = O) and amine (N–H) groups in amide (–CONH) linkage. The encapsulation efficiency of SPIO was higher than 57%, and it was not significantly affected by increasing the quantity of SPIO (Supplementary Figure 2); the weight percent of encapsulated SPIO in MF-MNB was 5.7 ± 0.54, 12.1 ± 1.22, 17.6 ± 1.39, 25.4 ± 2.13, and 31.2 ± 2.67wt%, respectively. In addition, as shown in the magnetization curve in Figure 2D, the magnetic property of MF-MNB was gradually increased to 22.1 emu/mg by increasing the SPIO weight percent to 17.6%; the high magnetic property may be attributed to the dense aggregation of SPIO in MF-MNB polymer shells. The results of these chemical and physical characterizations demonstrated the successful synthesis of MF-MNB.

### Drug Loading and FUS-triggered Drug Release Test

The potential of the MF-MNB as a drug carrier was evaluated by its DOX loading efficiency. As revealed in Supplementary Figure 3, the high drug loading efficiency of MN-MNB (more than 61.5%) was not obviously affected by the SPIO encapsulation until the SPIO weight percentage increased to 17.6%. However, when the SPIO encapsulation was higher than 17.6 wt%, the drug loading capacity dramatically decreased, and the formation of MNF-MNB was affected by the large amount of SPIO, resulting in structure destruction of some MF-MNBs (Supplementary Figure 4B).

**FIGURE 3:**
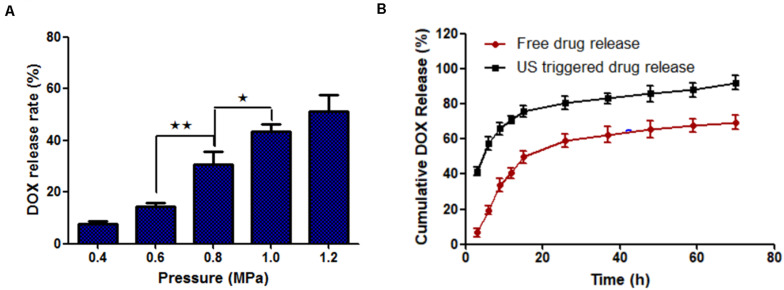
FUS-triggered DOX release from MF-MNB. **(A)** Drug release rate depends on different FUS sonication powers of 0.4, 0.6, 0.8, 1.0, and 1.2 MPa. **(B)** Time-dependent DOX release profile with/without FUS sonication (*p < 0.05, **p < 0.01).

The drug release rate of MF-MNB (SPIO 17.6 wt%) with FUS sonication was investigated to evaluate the effects of FUS sonication on drug release. As shown in Figure 3A, the drug release rate was increased upon FUS treatment. In particular, when the pressure of FUS increased to 0.8 MPa, the drug release rate was enhanced to almost twofold in 0.6 MPa, and the drug release rate was further increased to 41% when the pressure was 1 MPa. The MF-MNB after FUS sonication was observed with TEM (Supplementary Figure 5), with most of the MF-MNBs found to be destroyed or deformed, which could be due to the shear force from acoustic radiation force and cavitation effect (Cool et al., 2013). Moreover, the release of DOX from MF-MNB with/without FUS sonication was also evaluated for a 70-h period (Figure 3B). The results demonstrated that the release rate of DOX with FUS sonication was significantly enhanced when compared with that without FUS sonication, and approximately 92% of DOX was released in 2 days, while the MF-MNB without FUS sonication was less than 69% of the encapsulated DOX released. These results indicated that the DOX release from MF-MNB can be controllably triggered with FUS.

### *In vitro* US and MR Imaging Evaluation of MF-MNB

**FIGURE 4:**
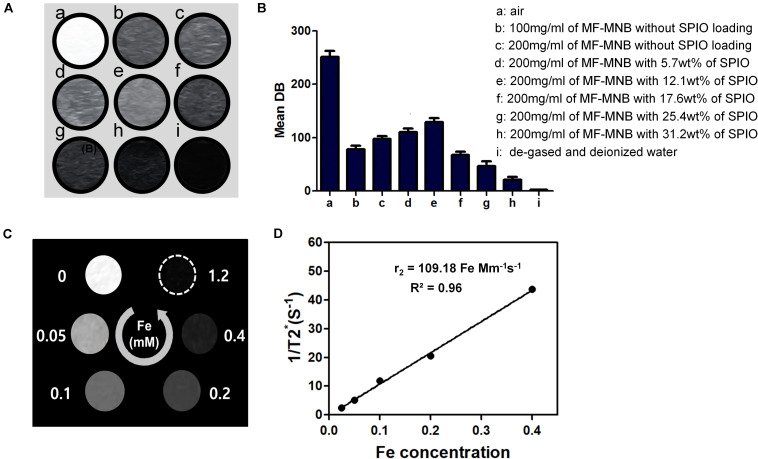
US and MR image of MF-MNB. **(A,B)** US image and US signal intensity of **(a)** air, **(b and c)** 100 mg/ml and 200 mg/ml of MF-MNB without SPIO loading, **(d to h)** 200 mg/ml of MF-MNB with different SPIO weight percent of 5.7, 12.1, 17.6, 25.4, and 31.2, and **(i)** degassed and deionized water. **(C)** T_2_-weighted MR image of MF-NRS in aqueous solution at different Fe concentration. **(D)** Relaxation rate *r*2 (1/T_2_) of MF-NRS at different as a function of Fe concentrations.

The capability of MF-MNB as US and MR contrast agent was evaluated via *in vitro* phantom test. As shown in a US imaging test (Figures 4A,B), degassed and deionized water were used as the positive and negative control. The MF-MNB exhibits obvious contrast-enhanced US signal effect, in which the US signal intensity was increased by increasing MF-MNB concentration. Moreover, the US signal was further gradually enhanced to 129 (gray scale value) by increasing the SPIO content to 17.6 wt%. However, when the SPIO content was over 17.6 wt%, the US signal intensity steeply decreased due to the inability to form the bubble-like structure as well as decreased encapsulated amount of perfluorocarbon gas. In addition, the MF-MNB enables it as a promising candidate for T_2_-weighted MR imaging due to the high magnetic property and excellent biocompatibility of MF-MNB. T_2_-weighted MR imaging data revealed that the developed MF-MNB could weaken signal intensity of the MR images with the increasing Fe concentration (Figure 4C). Furthermore, the relaxation rate (1/T_2_), also named as relaxivity r_2_, was assessed to quantitatively evaluate the MR contrast ability of the MF-MNB. As indicated in Figure 4D, the T_2_ relaxivity was calculated to be 109.18 mM^–1^ s^–1^, which is enough for further animal MR imaging.

### *In vitro* Cellular Uptake and Cytotoxicity

**FIGURE 5:**
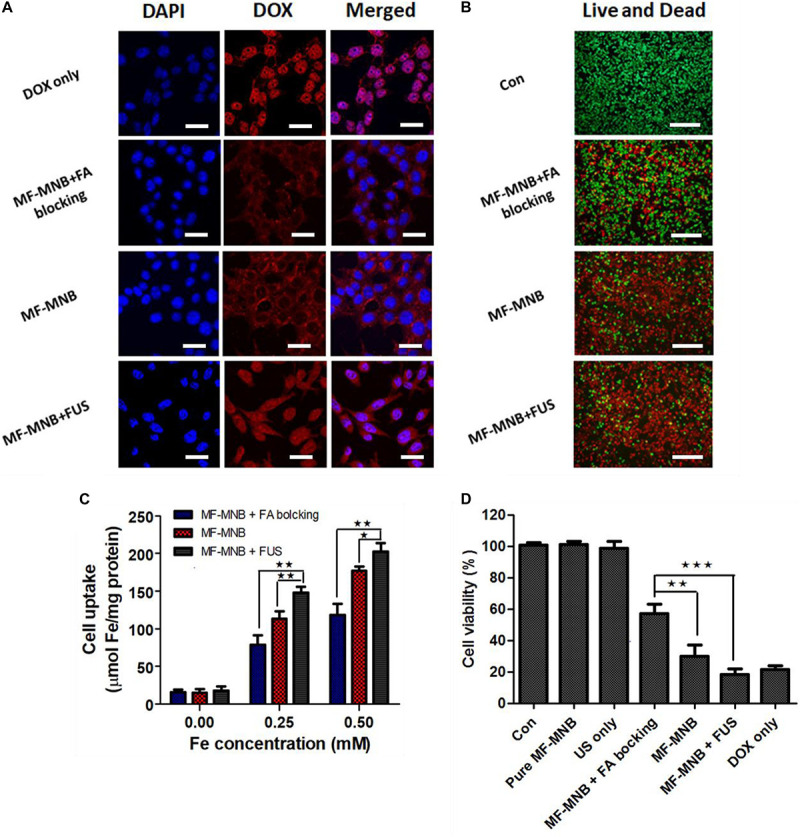
*In vitro* cell test results. **(A)** Confocal microscopy images of live 4T1 cells treated with free DOX, MF-MNB with FA receptor blocking, MF-MNB, and MF-NRS with FUS sonication (blue fluorescence is associated with DAPI and the red fluorescence shows DOX). All scale bars are 30 μm. **(B)** Fluorescence microscopy images of live and dead 4T1 cells treated with PBS, MF-MNB with FA receptor blocking, MF-MNB, and MF-NRS with FUS sonication (green fluorescence is associated with calcein AM and the red fluorescence shows PI). All scale bars are 200 μm. **(C)** Quantification of iron concentration on 4T1 cells after treatment with MF-MNB with FA receptor blocking, MF-MNB, and MF-MNB with FUS sonication. **(D)** Viability of 4T1 cells after incubation for 24 h with the same treatment with those in the fluorescence images (*p < 0.05, **p < 0.01, ***p < 0.001).

The subcellular localization of free DOX and MF-MNB in 4T1 cells and the FUS sonication-induced effect were observed using confocal microscope imaging (Figure 5A). Free DOX showed an obvious red fluorescence within cell nuclei, which may be due to the direct diffusion of small molecules like DOX into the cell. In contrast, 200-nm-sized MFMNBs have to undergo endocytosis process to enter the cells. Therefore, the DOX fluorescence from MF-MNB was mainly observed in the perinuclear cytoplasm. The MF-MNB-treated groups exhibited a much higher fluorescence signal when compared with the MF-MNB with FA blocking treatment groups. These results demonstrated that MF-MNB targeted the 4T1 tumor through FA receptor-mediated targeting. After FUS sonication, a strong red fluorescence accumulated in the cytoplasm and nucleus attributed to the enhanced cellular uptake by the sonoporation effect and the rapid drug release upon FUS exposure. In addition, the enhanced cellular uptake of MF-MNB by FA receptor-mediated targeting and FUS sonication was further evaluated by quantifying the intracellular iron accumulation. As shown in Figure 5C, the significant enhanced cellular internalization of MF-MNB occurred with the FA receptor targeting; the iron concentration in 4T1 cells with MF-MNB reached 178 μmol/mg of protein, while that in FA receptor-blocked 4T1 cells was only 120 μmol/mg of protein. Moreover, the cellular uptake can be further enhanced to 202 μmol/mg of protein by FUS sonication ascribed to the enhanced permeability of tumor cell membrane (Mehier-Humbert et al., 2005]. Finally, the cell cytotoxicity of various formations was further evaluated by live and dead cell detection and MTT assay. Figure 5B and Supplementary Figures 5, 6 show the fluorescence microscopy images of live and dead cells; no obvious dead (red) cells were observed in the pure MF-MNB or FUS exposure group, indicating that pure MF-MNB and FUS sonication for 3 min did not induce obvious cytotoxicity to the cancer cells. However, part of the cells found to be dead in MF-MNB with FA receptor blocking cells and the number of dead cells in MF-MNB group were obviously increased, which could be due to the enhanced cellular uptake of MF-MNB by FA ligand-receptor-mediated targeting. Then, the anticancer effect was further improved by FUS sonication. Moreover, the cytotoxicity was further evaluated with MTT assay, as shown in Figure 5D, and the cell viability had no obvious changes with pure MF-MNB or FUS sonication. MF-MNB showed an obvious heightened anticancer effect compared to MF-MNB with FA receptor blocking, and the cell viability was further decreased to 18.5% in 24 h with FUS sonication, attributed to the enhanced cellular uptake and rapid DOX release from MF-MNB. The cell viability was in accordance with the results from the live and dead cell detection test. All these results suggested that the fabricated MF-MNB could effectively kill the cancer cell with FA receptor-mediated tumor cell targeting and FUS sonication.

**FIGURE 6:**
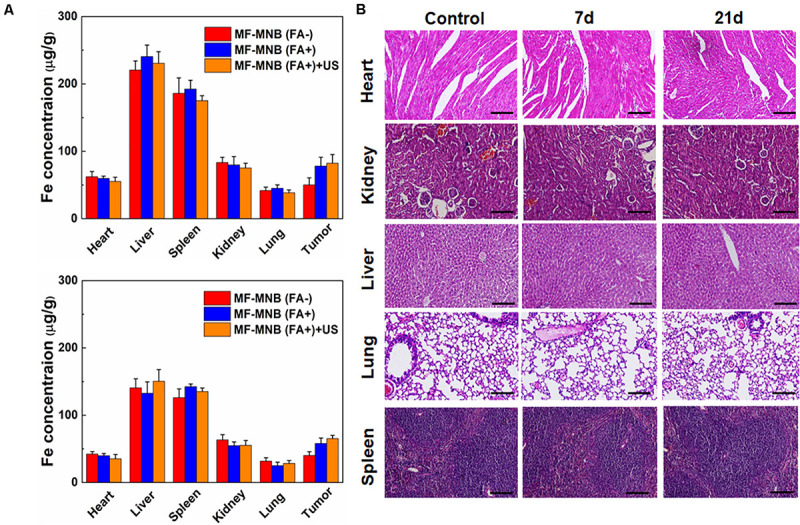
Biodistribution and pathological analysis. **(A)** Biodistribution of MF-MNB without FA modification, MF-MNB, and MF-MNB with FUS sonication for 3 min, determined by ICP-OES (*n* = 3), in major tissues of mice bearing 4T1 tumors after 4 and 24 h. **(B)** H&E-stained images of major organs from untreated healthy mice and treated mice with MF-MNB injection and FUS sonication taken 7 days and 21 days. No noticeable abnormality was observed in major organs (liver, spleen, kidney, heart, and lung).

### *In vivo* Targeted Biodistribution and Histological Analysis

In order to understand the *in vivo* targeted delivery of these NPs to the tumor sites, the 4T1 tumor-bearing mice were intravenously injected with MF-MNB with or without folic acid modification. US was also deployed to further enhance the deep penetration of the NPs inside the tumor. Then, the mice were sacrificed at 4 and 24 h to collect the major organs and subject to the ICP-OES analysis to evaluate the Fe amount in various major organs. As shown in Figure 6A, the MF-MNB NPs without FA indicated relatively low passive tumor accumulation in the tumor at both time points due to the EPR effect in cancerous tumors. In comparison, the accumulation of Fe was significantly increased for the mice after the injection of MF-MNB NPs coupled with FA. It revealed that FA modification could improve the targetability of the MF-MNB attributed to the high affinity of folic acid to the receptor on the 4T1 tumor cells. Furthermore, the US also exhibited slight facilitation to the uptake of MF-MNB NPs inside the tumors. It may be ascribed to the fact that the US could promote the permeability of vascular endothelial cells and ECM in tumor tissue, thus increasing the penetration distance of NPs inside the tumors. On the other hand, the injected NPs exhibited high accumulation reticuloendothelial systems (RES) such as, liver, spleen, and kidney at 4 h and then were gradually removed from these organs in 24 h.

Furthermore, we also carried out a histological analysis for the major organs from mice after the injection of MF-MNB for 7 and 21 days to evaluate the *in vivo* toxicity of the prepared NPs. As revealed in Figure 6B, the histological images indicated that no remarkable difference could be observed for organs of the control group and experiment groups. In addition, there were no obvious organ damage and inflammatory lesion in the major organs post-injection. It suggested that the prepared MF-MNB could not cause any obvious toxicity in biological applications.

## Discussion

In this presented study, we fabricated a novel MF-MNB for tumor-targeted US/MR imaging and FUS-triggered drug delivery. A biocompatible and biodegradable polymeric material of PLGA-PEG-FA was designed and synthesized because the copolymer may enable excellent loading capacity of hydrophobic and hydrophilic drugs or moieties (for example, SPIO, gold nanoparticles, or other biomedical moieties) (Hao et al., 2013). The synthesized MF-MNB exhibits excellent biocompatibility and stability in PBS buffer and DMEM as shown in Supplementary Figures 7, 8 with a hydrodynamic size of MF-MNB ranging from 91.7 nm to 459 nm, while the size range of the gaps in the leaky vasculature was reported as 600 nm to 800 nm. Furthermore, the PEG chain on the MF-MNB surface could form a flexible polymer brush layer that can prevent the absorption of opsonins (protein) on the MF-MNB surface, avoiding recognition by MPS, and thus prolong the circulation time. Therefore, the developed MF-MNB could efficiently pass through the leaky vasculature and accumulated in the tumor site.

Moreover, the presented MF-MNBs exhibits obvious enhanced cellular internalization and accumulation rate in the tumor site by the FA receptor-mediated tumor targeting, because the FA receptor (FR) is generally overexpressed in most types of tumors including ovarian, kidney, brain, breast, and liver (Maeng et al., 2010). However, before they can bind to the targeting tumor cells, the MF-MNBs also have to undergo the extravasation of blood vessels in tumors, penetrating deeply into the interstitium and crossing multiple cell layer processes together with various barriers, such as intratumoral pressure, shear force, binding-site effect, and multidrug resistance (Blanco et al., 2015; Miao et al., 2016). D.B. Kirpotin et al. reported that 100-nm-sized HER2-targeted liposomes accumulate to a higher extent in cancer cells than in macrophages, but do not achieve higher tumor concentrations (Kirpotin et al., 2006). The accumulation of MF-MNB with a mean diameter of 208 nm in tumor could also be limited. However, the aforementioned barriers could be properly overcome by employing FUS. The shear force and binding site effect could be overcome by enhancing the extravasation using the transient cavitation and acoustic radiation force generated by FUS sonication (Frenkel, 2008). Apfel and Holland (1991) calculated that US of 1 MHz can induce transient cavitation when the negative pressure is above 0.3 MPa, and the probability of cavitation occurrence increases as peak negative pressure increases and as frequency decreases. Moreover, acoustic streaming can be formed when the acoustic pressure and frequencies are on the order of MPa and MHz, which produce the translational force that pushes the nanocarriers against the blood vessel walls (Boissenot et al., 2016). In addition, the intratumoral pressure could also be overcome by acoustic streaming effect; the shear force created by FUS sonication can widen the intracellular space between endothelial cells and thus enhance the penetration of nanocarriers into solid tumor (Frenkel, 2008 and Couture et al., 2014). Consequently, the enhanced accumulation rate of MF-MNB in tumor by using FUS sonication (the results are shown in Figure 6A) is probably due to the transient cavitation and acoustic streaming effect as we used 925 kHz US with a peak negative pressure of 1 MPa.

In addition, the US imaging signal could be obviously enhanced by MF-MNB, in which the signal gradually increased with the increase in SPION loading quantity in MF-MNB shells. The US signal of the MF-MNB with 17.6 wt% SPIO loading was distinctly heightened more than 64% compared with MF-MNB without SPION loading because the densely imbedded SPIO in bubble shell leads to the MF-MNB not only being resonant with the US but also backscattering the US signal (Yang et al., 2009). The MF-MNB also exhibits promising MRI contrast effect with the T_2_ relaxivity of 109.18 mM^–1^ s^–1^ attributed to the densely aggregated SPIO in the MF-MNB bubble shell. Therefore, the biodistribution and accumulation rates of intravenously administered MF-MNB could be precisely monitored with dual-modal US/MR imaging, in which the tumor progress after treatment could also be observed. These results will be absolutely meaningful to further design the treatment strategy.

Furthermore, after the proper accumulation of MF-MNB in the tumor site, the triggering DOX release with FUS sonication should be performed to lead the drug concentration in the tumor to rapidly reach the lethal dosage and thus effectively kill the cancer cells and avoid multidrug resistance, which could occur when the tumor cells are frequently exposed to sublethal drug dosage (Gottesman et al., 2002). The rapid DOX release could be achieved by the disruption or deformation of MF-MNB with the shear force from acoustic radiation force or acoustic cavitation, and there will be no thermal mechanisms due to the FUS exposure conducted in this study at about 2.1% duty cycles (the total operation time of FUS is about 21 ms in 1 s, and 979 ms is off). In addition, FUS exposure could further improve the dispersion of released drugs throughout the tumor cells and tissues attributed to the enhanced motion of fluid in the vicinity of the drug and the target tumor tissues by acoustic radiation force and transient cavitation bubble oscillations (Elkhodiry et al., 2016), thus enhancing the therapeutic efficiency.

## Conclusion

In summary, we fabricated a folate receptor-targeted MF-MNB for US and MR imaging and FUS-triggered drug delivery. The MF-MNB can stably entrap more than 17.6 wt% of SPIO nanoparticles into the polymer shell and load drug with an efficiency of more than 64% into the core. Moreover, the MF-MNB exhibits a promising US and MR imaging contrast effect due to the densely aggregated SPIO nanoparticles in the bubble shell, providing excellent magnetic property and enhancing US signal. In addition, an almost threefold faster drug release time and 22% increased DOX release from MF-MNB in 2 days were achieved by FUS triggering. Furthermore, the cellular uptake was enhanced more than 100% by FA receptor targeting and FUS sonication. Moreover, the cell cytotoxicity of MF-MNB with FA ligand and FUS sonication was evaluated with MTT assay, and a significantly decreased cell viability of 18.5% in 24 h was achieved. In addition, the accumulation of MF-MNB was enhanced by FA ligand-receptor-mediated tumor targeting and FUS sonication. Hence, we believe that the fabricated MF-MNB is a promising drug nanocarrier for US/MR imaging-guided and FUS-triggered drug delivery for efficient cancer treatment.

## Data Availability Statement

All datasets presented in this study are included in the manuscript/Supplementary Material.

## Author Contributions

ZJ investigation, validation, and writing the manuscript. JLC, PD, SJ, MJ, and HT investigation and validation. WSJ and WR writing review and editing. SZ funding acquisition, project administration, and writing review and editing. All authors contributed to the article and approved the submitted version.

## Conflict of Interest

The authors declare that the research was conducted in the absence of any commercial or financial relationships that could be construed as a potential conflict of interest.

## Supplementary Material

Supplementary Figure 1 ^1^HNMR spectrum of PLGA-PEG-FA polymer.

Supplementary Figure 2 SPIO encapsulation efficiency with different weight percent of SPIO to PLGA-PEG-FA polymer.

Supplementary Figure 3 DOX loading efficiency in MF-MNB with different weight percent of SPIO in MF-MNB.

Supplementary Figure 4 TEM images of MF-MNB with **(A)** 5.7wt% of SPIO and **(B)** 25.4wt% of SPIO.

Supplementary Figure 5 TEM image of MF-MNB after treated with FUS for 3 min.

Supplementary Figure 6 Fluorescence microscopy images of **(A)** live and dead 4T1 cells treated with FUS sonication for 3 min, **(B)** pure MF-MNB and **(C)** free DOX (green fluorescence is associated with Calcein AM and the red fluorescence shows PI). All scale bars are 200 μm.

Supplementary Figure 7 Biocompatibility test of MF-MNB. **(A,B)** Viability of NIH3T3 and 4T1 cells incubated for 24 h with DOX unloaded MF-MNB.

Supplementary Figure 8 Stability test of MF-MNB in PBS buffer (pH 7.4), black line is the size distribution of MF-MNB in PBS at the time of 0 h, red line is the size distribution of MF-MNB at the time of 24 h.
