# Supplementary material for: Tumor Targeted Multifunctional Magnetic Nanobubbles for MR/US Dual Imaging and Focused Ultrasound Triggered Drug Delivery
Source: Front Bioeng Biotechnol. 2020 Dec 7;8:586874. doi: 10.3389/fbioe.2020.586874 (PMC7750502; doi:10.3389/fbioe.2020.586874)
Supplement: Supplementary Figure 1 — 1HNMR spectrum of PLGA-PEG-FA polymer. [file Data_Sheet_1.pdf]

Supplement material

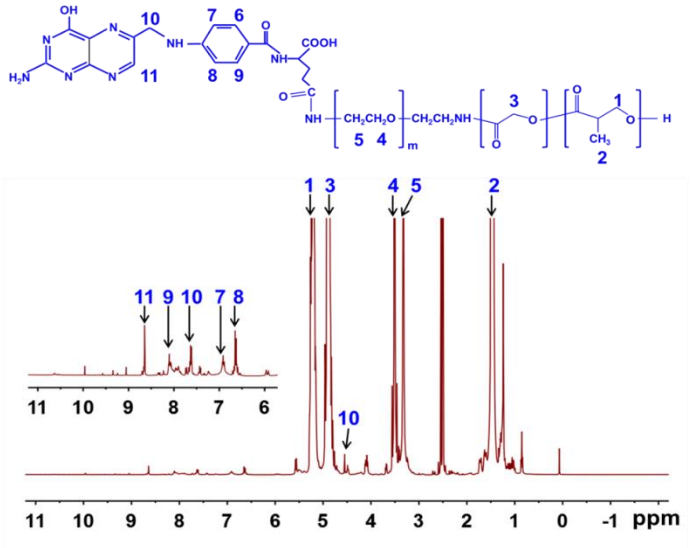

Figure S1. ^{1}HNMR spectrum of PLGA-PEG-FA polymer.

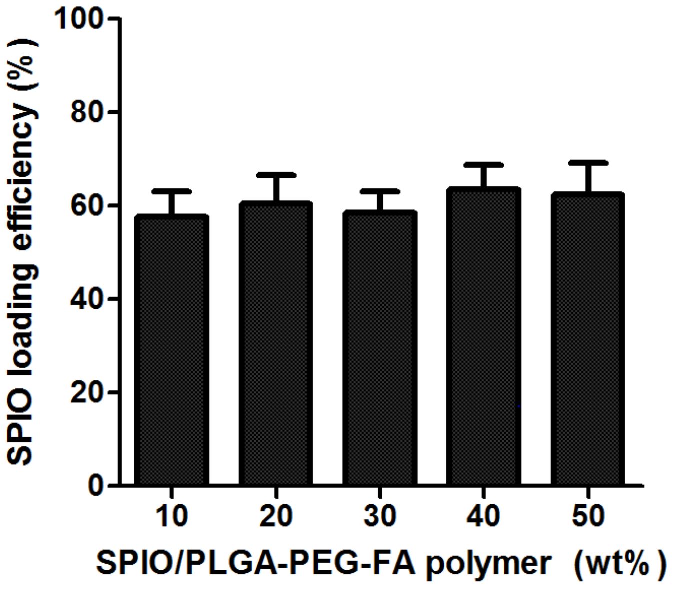

Figure S2. SPIO encapsulation efficiency with different weight percent of SPIO to PLGA-PEG-FA polymer.

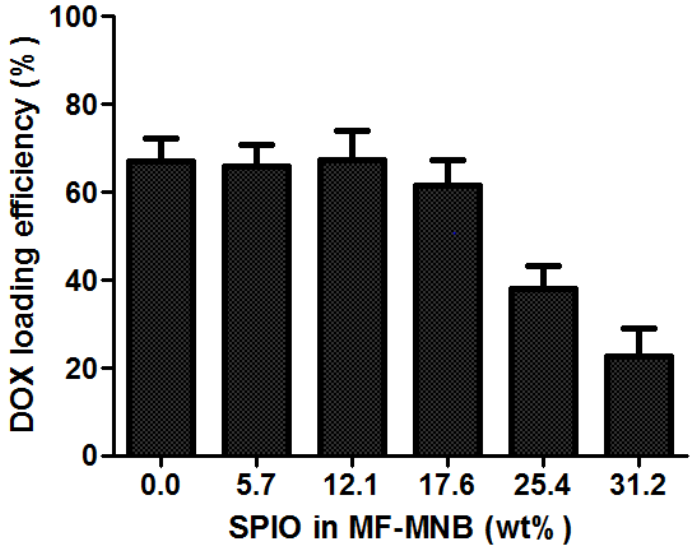

Figure S3. DOX loading efficiency in MF-MNB with different weight percent of SPIO in MF-MNB.

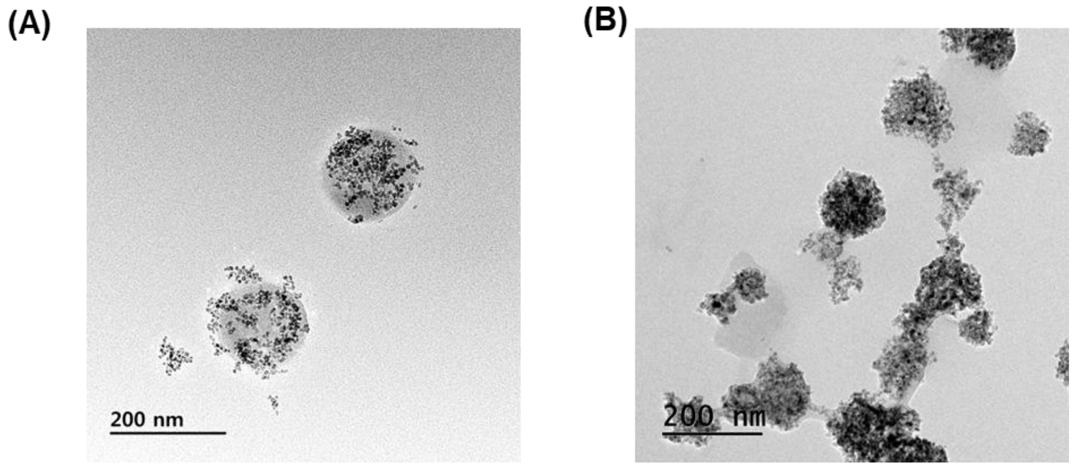

Figure S4. TEM images of MF-MNB with (A) 5.7wt% of SPIO and (B) 25.4wt% of SPIO.

μm.

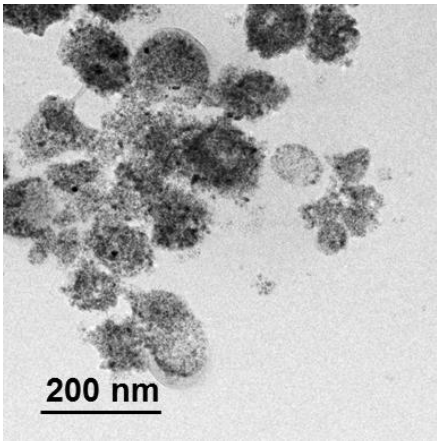

Figure S5. TEM image of MF-MNB after teated with FUS for 3min.

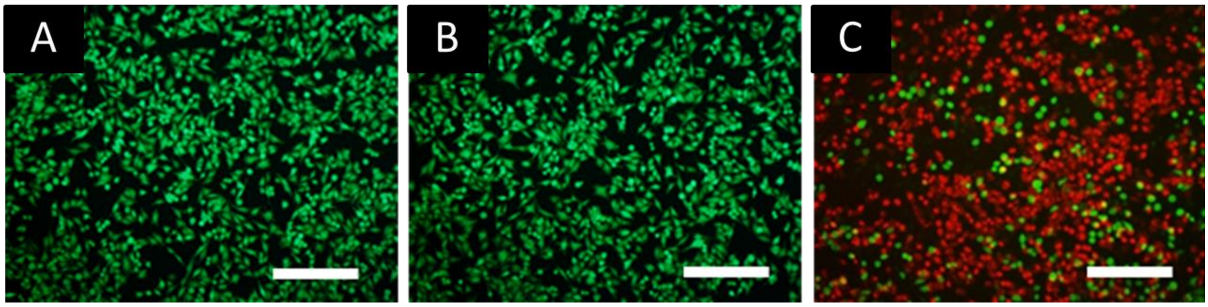

Figure S6 Fluorescence microscopy images of (A) live and dead 4T1 cells treated with FUS sonication for 3min, (B) pure MF-MNB and (C) free DOX (green fluorescence is associated with Calcein AM and the red fluorescence shows PI). All scale bars are 200 μm.

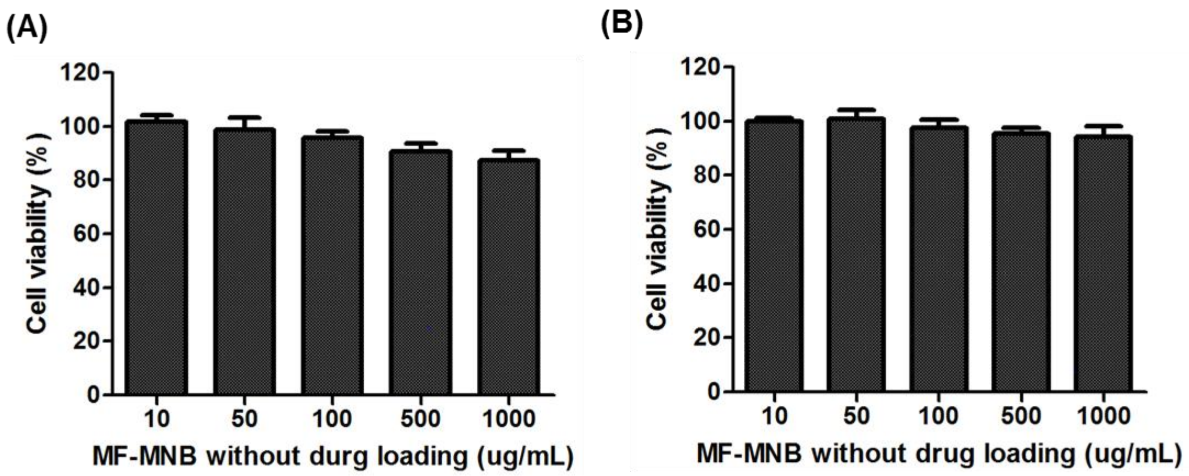

Figure S7. Biocompatibility test of MF-MNB. (A) and (B) Viability of NIH3T3 and 4T1 cells incubated for 24 h with DOX unloaded MF-MNB.

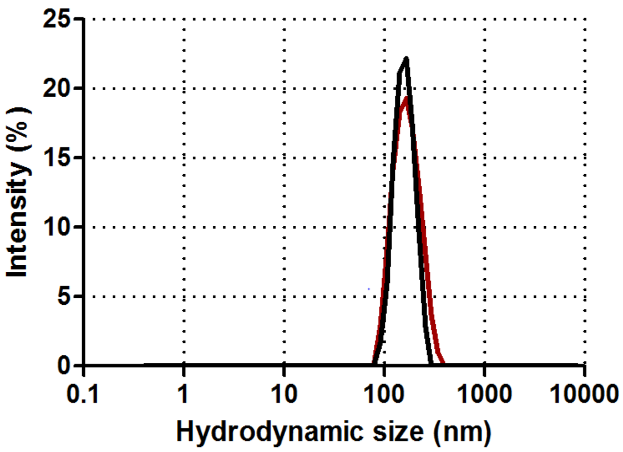

Figure S8. Stability test of MF-MNB in PBS buffer (pH 7.4), black line is the size distribution of MF-MNB in PBS at the time of 0h, red line is the size distribution of MF-MNB at the time of 24h.
